# Discovery and validation of small molecule stabilizers of mutant triose phosphate isomerase (TPI) as potential lead candidates for TPI deficiency

**DOI:** 10.1016/j.slasd.2025.100278

**Published:** 2025-09-16

**Authors:** Laura L. Vollmer, Presley Roberts, Samantha L. Eicher, Marta Wołosowicz, Priyal Patel, Joseph R. Figura, Ella R. Donahue, Josh Berkowitz, Dillon Gavlock, Peter Wipf, Matt LaPorte, Steven J. Mullett, Amelle Shillington, Gregg E. Homanics, Michael J. Palladino, Andreas Vogt

**Affiliations:** a Department of Computational & Systems Biology, Organ Pathobiology and Therapeutics Institute, University of Pittsburgh School of Medicine, Pittsburgh, PA, 15219, USA; b Department of Pharmacology & Chemical Biology, University of Pittsburgh School of Medicine, Pittsburgh, PA, 15261, USA; c Pittsburgh Institute for Neurodegenerative Diseases, University of Pittsburgh School of Medicine, Pittsburgh, PA, 15261, USA; d Department of Chemistry, University of Pittsburgh, Pittsburgh, PA, 15260, USA; e Department of Human Genetics, Cincinnati Children’s Hospital Medical Center, Department of Pediatrics, University of Cincinnati College of Medicine, Cincinnati, OH, 45229, USA; f Department of Anesthesiology and Perioperative Medicine, University of Pittsburgh, Pittsburgh, PA, 15261, USA

**Keywords:** Triose phosphate isomerase, TPI deficiency, Metabolic disease, Early childhood disease, High-content screening

## Abstract

*Triosephosphate Isomerase* deficiency (TPI-Df) is a devastating untreatable childhood metabolic disease resulting in anemia, severe locomotor impairment, and premature death. Numerous single amino acid substitutions in *TPI* are pathogenic and result in rapidly progressing multisystem disease. Importantly, all known pathogenic TPI-Df mutations result in a protein that retains function, and pathogenesis is known to result from decreased steady state levels of the functioning protein. There are no small molecule therapies for TPI-Df; current treatments are limited to symptomatic support and dietary interventions. We reasoned that a phenotypic screen was most appropriate to capture agents that stabilize mutant TPI and developed a human cellular TPI-Df assay based on a cellular model of the “common” TPI^E105D^ mutant protein fused with a GFP and a fluorescent ROS biosensor. The assay was implemented for high-content, high-throughput imaging, optimized to full HTS standards, and used to screen a 2,560 compound pilot library and the 220,700 compound NIH MLSMR compound collection to identify candidate compounds for development into small molecule TPI-Df therapies. Hits were validated in dose-response, TPI-Df patient cells, and various orthogonal assays. Limited SAR revealed three promising compound series, which were evaluated for potential mechanisms of action. The lead series had previously been identified as inducers of HIF1 alpha, spawning a novel hypothesis that HIF1 alpha activation might be a potential avenue to treat TPI-Df patients. A lead molecule was moved into preliminary mouse studies to evaluate pharmacokinetics and tissue distribution and was shown to be moderately brain-penetrant. The lead compound is now positioned for target identification studies and efficacy testing in vivo TPI Df models, including a newly validated mouse model.

## 1. Introduction

Inborn errors in metabolism often present early in life and are particularly heinous diseases when no curative treatment is available. TPI Deficiency (TPI-Df) is such a disease, owing to impaired glycolysis that results from various pathogenic mutations in the *TPI1* gene that lead to expression of mutant proteins with reduced stability, which is the root cause of TPI-Df. Patients homozygous for the common *TPI^E105D^* mutation are typically diagnosed at 1–2 years of life after hemolytic crisis requires hospitalization and a blood transfusion. The typical disease progression involves anemia, delayed and missed developmental milestones, and progressive neuromuscular impairment that hinders locomotion and increases dependence upon medical support leading to an early death. Allelic combinations that are more or less severe are rare but have been reported [1,2]. Treatments for TPI-Df are currently limited to symptomatic support and dietary interventions. Patients are monitored hematologically and receive periodic blood transfusions. Some patients have received bone marrow transplants that appear to fully correct the anemia, however it is not known whether there are additional benefits to this treatment.

TPI is an isomerase that interconverts dihydroxyacetone phosphate (DHAP) and glyceraldehyde-3-phosphate (G3P). Pathogenic mutations in *TPI1* impair glycolytic efficiency and result in elevated DHAP, an unstable 3-carbon intermediate. DHAP non-catalytically degrades to methylglyoxal (MGO), a highly reactive α-di-carbonyl compound that causes widespread proteolytic toxicity. Although glyoxalases exist to destroy MGO and limit their toxicity, severe deficiency of TPI is thought to overwhelm this system resulting in increases in MGO. It is currently not known how much of TPI-Df disease pathogenesis results from bio-energetic impairment and increased reliance upon mitochondrial energy production or from MGO toxicity. There is considerable evidence that DHAP accumulation contributes to disease pathogenesis through the elevation of methylglyoxal (MGO) [3,4]. MGO is a highly reactive α-di-carbonyl that is almost certainly a potent cellular toxin. MGO has been shown to create covalent adducts with DNA and proteins [5] but it has also been observed that MGO induces non-enzymatic post--translational modifications in TPI [4]. Such a process could contribute to the rapidly progressing nature of this disease. As there are currently no treatments for TPI-Df, it is recommended that patients be placed upon a diet low in carbohydrates with increased calories from fats and proteins, such as a ketogenic diet, to decrease glycolytic flux and the production of MGO.

TPI-Df results from a severe decrease in TPI protein and activity [6-8]. Interestingly, pathogenic *TPI1* mutations produce a protein that retains high levels of activity but the protein’s turnover is increased, leading to low steady state levels. Thus, small molecules that increase steady state TPI levels could be a viable therapy for TPI-Df. We reasoned that a phenotypic screen for cellular TPI expression levels was most appropriate to capture agents that increase mutant TPI and developed a human cellular TPI-Df assay based on a cellular model of the “common” TPI^E105D^ mutant protein fused with a GFP and a fluorescent ROS biosensor that allows TPI levels to be conveniently monitored [9]. Using this human cellular model several small molecule compound libraries were sucessfully screened, which led to the identification of compounds that elevated TPI and were validated as proof of concept in patient cells [2,9]. This manuscript describes the development and execution of this image-based screening method to HTS standards for the identification of novel TPI-Df therapeutics, together with secondary assays that resulted in the discovery of several agents positioned to move into animal models of TPI-Df.

## 2. Materials and methods

### Generation and culturing HeLa *TPI^E105D-GFP-HyPerRed^* (*TPI^E105D-GFP-HR^)* cells.

HeLa cells stably expressing TPI-E105D-eGFP were generated as previously described [9]. A construct with the *EF1* promoter driving HyperRed protein expression was generated using a *HyperRed* clone (Addgene # 48,249 [10], and standard cloning techniques. The *Ef1-HyperRed* lentivirus was made commercially (Alstem Bio). Lenti-virus transduction of *TPI-E105D-eGFP* lines was performed to generate stable cells co-expressing both GFP-tagged mutant TPI (E105D variant) and the HyperRed ROS biosensor. Cells were sorted by flow cytometry to collect a population of cells that expressed intermediate levels of GFP.

### TPI-Df patient cells.

Patient fibroblasts were obtained from a male TPI deficiency patient via skin punch and fibroblasts were grown in culture similar to previously reported methods [2,11] The cells were de-identified, confirmed to be *TPI-E105D* homozygous by standard PCR and sequencing, and are known as FB303. Fibroblasts were cultured using standard methods (37 °C, 5 % CO2) in complete media (DMEM with 20 % fetal bovine serum (FBS), 100 U penicillin/100 μg/mL streptomycin (Lonza), 2 mM l-glutamine (Gibco) and supplemental non-essential amino acids (Gibco). For experiments, medium was changed to 10 % FBS.

### HTS assay development.

HeLa *TPI^E105D-GFP-HR^* cells were subjected to a formal assay development paradigm according to the NIH assay guidance manual [12]. Specifically, we evaluated optimal cell seeding, DMSO tolerance, luminespib positive control dose-response and timing, and, for live cell assays, signal stability in the presence of Hoechst 33324 (not shown; cf [9]). Briefly, cells were plated in collagen-coated 384 well microplates (Revvity PhenoPlates) and allowed to attach overnight. Cells were treated with test agents for various lengths of time, nuclei stained with Hoechst 33342, and imaged on an OPERA Phenix Plus high content reader (Revvity, inc., Waltham, MA) with a 20x air objective and laser lines and emission filters for Hoechst/DAPI (ex405/em435–480 nm), GFP (ex488/em500–550 nm), and Cy3 (ex561/em570–630 nm). Images were archived and analyzed in Harmony 5.1. (Revvity) for GFP levels, cell numbers, nuclear morphology, and ROS levels. The final conditions of the optimized assay were 4000 cells plated, a 48 h exposure to test agents in 1 % DMSO, and for live cells, an assay window of up to 6 h in the presence of Hoechst 33342. Cells were subsequently fixed with 4 % formaldehyde for possible re-imaging.

### Compound libraries.

The Cumbre library is a pilot compound collection of ~2500 diverse small molecules at the Organ Pathobiology and Therapeutics Institute (OPTIn, formerly UPDDI) to gauge assay performance under screening conditions, and to establish and validate a secondary assay paradigm. The Molecular Libraries Small Molecule Repository (MLSMR) is a 220,700 member compound collection that was assembled by the NIH through the Molecular Libraries Roadmap Initiative as part of its mission to enable the use of compound screens in biomedical research [13]. This library is now highly annotated in Pub-Chem, and based on our experience has good compound availability for repurchase of authentic material in follow-on assays. All libraries are maintained as assay-ready daughter plates at the OPTIn under temperature and humidity-controlled conditions as recommended [14,15].

### Primary HTS.

A total of ~225,000 compounds were tested at a single concentration of 10 μM. Daughter plates containing 2 μL 1 mM test agents in DMSO were reconstituted in 63 μl growth medium to a final concentration of 30 μM in 3 % DMSO. To each treatment plate positive controls (750 nM luminespib) were added to columns 2 and 23 and negative controls (3 % DMSO) were added to columns 1 and 24 (*n* = 32 each) with an electronic multichannel pipettor. Plates were centrifuged at 50 x g for one minute, and 15 μL transferred to assay plates with a Janus MDT for final concentrations of 10 μM (1 % DMSO). After 48 h at 37 °C, cells were stained live with Hoechst 33342 and imaged as described above. Cells were then fixed with 4 % formaldehyde, washed with a Biotek EL-405 plate washer and stored in PBS at 4 °C for further analysis. All reagent additions were either with a Biotek Microflo or Thermo Fisher Multidrop bulk liquid dispenser.

### Initial hit selection.

HCS screening data were uploaded to Spotfire (Ver 14.0.2, Revvity) for analysis. A hit selection paradigm that included gates for expression of TPI-GFP, cell densities, nuclear morphology, and ROS levels was established to identify initial hits. Archived scan images of selected hits were then subjected to visual inspection. Compounds that caused clear and unambiguous elevation of GFP, no cellular or nuclear morphology changes, and no visibly elevated ROS were prioritized for secondary assays. Also prioritized were hits that were structurally similar or had analogs in the library that were devoid of activity. The final list of compounds was subjected to cheminformatics filtering for compounds with favorable “drug-like” physicochemical properties using the FAFDrugs4 server at https://fafdrugs4.rpbs.univ-paris-diderot.fr/ [16]. Specifically, this included no pan assay interference compounds (PAINS A,B,C) or toxicity flags, no Lipinski violations, and no potential reactivity or promiscuity using the lead-like filter in the Eli-Lilly MedChem Rules toolkit used for open drug discovery projects [17] and the Pfizer 3/75 [18] and GSK 4/400 [19] physchem rules, which flag compounds for potential reactivity and liabilities in future optimization studies.

### Dose-response confirmation.

Hits from the high-throughput screens that passed above selection citeria were obtained from commercial vendors as powders and reconstituted to 10 mM DMSO stock solutions. Ten-point dose-response gradients (in triplicate) were created by serially diluting 25 μL of 10 mM stocks into 25 μL DMSO with an Agilent Bravo liquid handler. Multiple sets of daughter plates were stamped out by transferring 2 μL stock solution to fresh microplates. Plates were sealed and stored at −20 °C. For experiments, cells were plated into thin bottom 384-well collagen-coated Phenoplates and allowed to adhere overnight in a humidified 37 °C, 5 % CO2 incubator. The next day, compound daughter plates were thawed and reconstituted in 63 μL medium creating a 3X treatment plate. Positive and negative controls were added to the treatment plates (*n* = 32 each), for a final concentration range of 100 μM – 200 nM (1 % DMSO). Compounds that confirmed in dose-response were repurchased from Molport (Riga, Latvia) and structure and purity confirmed by LC/MS and/or ^1^H NMR. Selected agents were resynthesized at gram scale by Enamine US Inc. (Monmouth Jct., NJ). A list of compounds used for follow-up studies is presented in Supplemental Table 1 and Supplemental Table 2. For HeLa cells, incubation, processing steps, fixation, imaging, and analysis are described in the HTS section; for immunofluorescence studies in TPI-Df patient cells, see section below.

### General LC-MS separation/analysis conditions.

Identity and purity of repurchased compounds mostly relied on analytical data (LC-MS/NMR) supplied by vendors. Where vendor information was not available, and for resynthesized material, purity was assessed in-house using an Agilent Technologies 1260 Infinity II LC at 220 nm UV absorption (Waters XBridge BEH C18 2.1 × 50 mm, 2.5 μm). A 10 min gradient elution with a MeCN/H_2_O/MeOH mobile phase containing 0.1 % formic acid at a flow rate of 500 μL/min was used with an eluent ratio from 3:92:5 at 0–0.5 min to 93:2:5 at 4.0 min, back to 3:92:5 from 6.0 to 7.5 min. Samples were analyzed by total ion chromatogram (TIC, positive ion mode) and UV (220 nm, 254 nm) detection, and confirmed identity and purity.

### Immunofluorescence in TPI-Df patient cells.

Patient fibroblasts were plated in collagen-coated Revvity Phenoplates, allowed to attach over-night, and treated with test agents in 10 point, two-fold gradients. Forty-eight hours after treatment, cells were fixed with 4 % formaldehyde for 15 min at RT. Cells were permeabilized with 15 μL blocking buffer (0.3 % Triton X-100 and 1 % BSA in PBS, 0.45 μm filtered) containing 10 μg/ml Hoechst 33342 for one hour at RT, followed by addition of 15 μL of primary antibody (1:1000 Rabbit poly PA5–21583 WH3264660A (Invitrogen)) overnight at 4 °C. Plates were washed three times with PBS and incubated with 1:500 Cy3-conjugated AffiniPure Donkey Anti-Rabbit IgG (*H* + *L*) secondary antibody (Jackson Immunoresearch 711–165–152) in blocking buffer for 45 min. at RT. Plates were washed with PBS, sealed and stored at 4 °C until analysis.

### Western Blotting.

FB303 patient fibroblasts [E105D/E105D] were treated with MolPort-000–442–846 (1 μM, 3 μM, or 10 μM), Molport 002–877–424 (5 μM and 50 μM), Molport 047–151–201 (10 μM and 50 μM), or DMSO (0.1 %–1 %, Sigma Aldrich), trypsinized (0.05 % for 5 min), pelleted, resuspended in RIPA buffer with protease inhibitors (PMSF (100 μM), Leupeptin (1 μg/μL), Pepstatin A (0.5 μg/μL) and pulse sonicated. Protein concentrations were determined using a BCA assay (Pierce). Immunoblotting was performed on whole protein cell lysates following the addition of an equal volume of 2 × SDS PAGE sample buffer (4 % SDS, 4 % β-mercaptoethanol, 130 mM Tris–HCl pH 6.8, 20 % glycerol). Proteins were resolved by SDS-PAGE (12 %), transferred onto 0.45 μm PVDF membrane. The blots were blocked in Odyssey Blocking Buffer (Licor) and incubated with anti-TPI primary antibody (1:1000 Rabbit polyclonal PA5–21583 WH3264660A (Invitrogen) or anti-beta-tubulin (1:1000; mouse polyclonal E7-C; Developmental Studies Hy-bridoma Bank) diluted in Odyssey Blocking Buffer (Licor). Following washes in PBST, the blots were incubated with anti-mouse-IR800 (Fisher Scientific) and anti-rabbit-IR680 (Molecular Probes) both diluted to 1:20,000 in 0.1 % Tween 20 blocking buffer. Blots were washed in PBST and developed using Odyssey Infrared Imaging System. Quantification of the scanned images was performed digitally using the Image Studio Ver 5.2 software. TPI levels were normalized to beta tubulin loading control, and further normalized to vehicle control.

### Mass Spectrometry.

S-Trap trypsin digestion. Protein lysates were prepared in 50 mM TEAB with 5 % SDS. 5 or 10 μg total protein as verified by BCA assay were digested on a micro-S-Trap (Protifi, Fairport NY) as per manufacturer instructions. The resultant tryptic peptides were extracted with 70 % acetonitrile (MeCN/5 % formic acid (FA), vacuum dried, and re-constituted in 20 μL 3 %MeCN/0.1 % FA.

### LC—HRMS Data Acquisition.

LC-MS/MS analysis was carried out using a nanoElute2 UHPLC system (Bruker Daltonics, Bremen, Germany) coupled to the timsTOF Pro2 mass spectrometer mass spectrometer (Bruker Daltonics), using a CaptiveSpray nanoelectrospray (Bruker Daltonics). Two μL, roughly 200 ng, of peptide digest was loaded on a capillary C18 column (25 cm length, 75 μm inner diameter, 1.6 μm particle size, 120 Å pore size; IonOpticks Aurora Gen3, Fitzroy, VIC, AUS). Peptides were separated at 55 °C using a 60 min gradient at a flow rate of 300 nL/min (mobile phase A (MPA): 0.1 %; mobile phase B (MPB): 0.1 % FA in MeCN. A linear gradient of 2–35 % MPB was applied for 60 min, followed by a 5 min wash at 95 % MFB before equilibrating the column at 2 % MFB for 6 min.

The Bruker timsTOF Pro2 was operated in data-dependent PASEF mode collecting full scan mass spectra from 100 to 1700 m*/z*. Ion mobility resolution was set to 0.60–1.60 V⋅s/cm over a ramp time of 100 ms. Data-dependent acquisition was performed using 10 PASEF MS/MS scans per 1.1 s cycle. Active exclusion time window was set to 0.4 min, and the intensity threshold for MS/MS fragmentation was set to 2.5e4 while low *m/z* and singly charged ions were excluded from PASEF precursor selection. MS/MS spectra were acquired via ramped collision energy as function of ion mobility.

### Data Analysis.

IM/MS raw files were processed using MSFragger (ver3.6). MS/MS spectra were searched against the OpenProt RNA transcriptome database consisting of alternative protein sequences resulting from all RNA transcripts reported by both Ensembl and NCBI RefSeq (https://openprot.org) The search parameters allowed for a 10 ppm precursor ion mass error, 20 ppm on fragment ion masses and a maximum 2 missed cleavages. Carbamidomethylation of cysteine was set as a fixed modification. Oxidization of methionine and acetylation of protein N-termini were set as variable modifications. False discovery rate (FDR) was capped at 1 % prior to data export of raw peak areas.

### Proteasome-Glo Trypsin-Like Cell-Based Assay.

FB303 patient fibroblasts [E105D/E105D] were trypsinized (0.05 % for 5 min), counted, and plated in white-walled optical bottom 96-well plates at a confluency of 5000 cells per well, and wells were filled to a total volume of 100 μL each with 10 % FBS DMEM. Cells were placed in a 37 °C, 5 % CO_2_ incubator for twenty-four hours to allow the fibroblasts to adhere to the wells. After overnight incubation, old media was removed and cells (in technical replicates) were treated with 100 μL of the following treatments: vehicle control (1 % DMSO), Molport 002–877–424 (50 μM), Molport 047–151–201 (50 μM), and MolPort-000–442–846 (10 μM). Treated cells were placed back in the incubator for two hours. During this incubation, Luciferin Detection Reagent was reconstituted in Proteasome-Glo Cell-Based Buffer according to manufacturer’s instructions (Promega). The Luciferin Detection Reagent was allowed to stand at room temperature for thirty minutes prior to use to remove any contaminating free aminoluciferin and decrease background luminescence. Following incubation, 100 μL of the Proteasome-Glo Cell-Based reagent was added to each well using a multi-channel pipette. Contents of the plate were mixed on an orbital shaker at 700 rpm for two minutes. The plate was incubated at room temperature for ten minutes, then placed into a SpectraMax iD5 microplate reader. Luminescence was measured every ten minutes for an hour and forty minutes at 22 °C.

### Statistical analysis.

Unless otherwise noted, data from multiple independent biological repeats were analyzed by one-way ANOVA with Holm-Šídák’s multiple comparisons test in Graph Pad Prism (Ver 10). p-values <0.05 were considered significant.

### Iron-dependence.

FB303 patient fibroblasts in 384 well microplates were treated and processed for immunofluorescence staining with anti-TPI antibody as described, except that one set of treatment solutions was supplemented with 100 μM ferrous chloride (FeCl_2,_ Sigma).

Mouse pharmacokinetics (PK) Mouse PK studies were performed exactly as described in [18-20] (except that Molport 002–877–424 was the test compound at 10 mg/kg i.p.) by Touchstone Biosciences (Ply-mouth Meeting, PA), following the National Research Council guide for the care and use of laboratory animals to ensure proper care, use, and human treatment of the animals used in the study, adhering to generally accepted procedures for animal housing, dosing and blood sampling, and following AVMA guidelines for euthanasia. All procedures were approved by an institutional review panel.

### PK data Analysis.

Plasma concentrations were measured as described [20] to determine a concentration vs. time profile. The area under the plasma concentration vs time curve (AUC) was calculated using the linear trapezoidal method. Fitting of the data to obtain pharmacokinetic parameters was carried out using non-compartmental analysis. Key PK parameters reported following i.p. administration are as follows: terminal half-life t_1/2_, initial plasma concentration C_0_, area under the plasma concentration vs. time curve AUC, volume of distribution at steady-state Vss, total plasma clearance CLp, and mean residence time MRT. All parameters are expressed for individual animals as well as mean, standard deviation, and coefficient of variation.

### Tissue distribution Study.

#### Animal Care.

All the animals were housed at the University of Pittsburgh under the supervision of the Department of Laboratory Animal Resources under protocols approved by the Institutional Animal Care and Use of Committee (IACUC) and in compliance with the National Institutes of Health guide for the Care and Use of Laboratory Animals. Animals were placed in individually ventilated cages under specific pathogenic free conditions with 12-h light/dark cycles with food and water available ad libitum.

### Experimental design.

Male C57BL/6 J (Jackson Laboratories) wild type mice (age: 40–60 days) weighing 25–30 g, were used for the tissue distribution study. Mice were randomly assigned in two groups: blank (*n* = 1) and treatment. The treatment group received 20 mg/kg compound 424 intraperitoneally (*n* = 15; 3 animals per time point i.e., 0.5, 1, 2, 4, and 8 h). Molport 002–877–424 was formulated in a vehicle consisting of 20 % DMSO, 50 % PEG300, and 30 % sterile water. At each time point animals were euthanized via isoflurane overdose. Blood was collected in ETDA-coated tubes at each time points and centrifuged at 15,000 x g for 5 min. The separated plasma was stored at −80 °C. Following blood collection, liver, kidney, skeletal muscle and brain were isolated, rinsed in normal saline and approximately 100 mg of tissues were snap-frozen and stored at −80 °C. All plasma and tissue samples were shipped on dry ice to Touchstone Biosciences for further analysis. Sample preparation and analysis was performed at Touchstone Biosciences exactly as described in [20].

## 3. Results

### Assay Development.

**Fig. 1.**
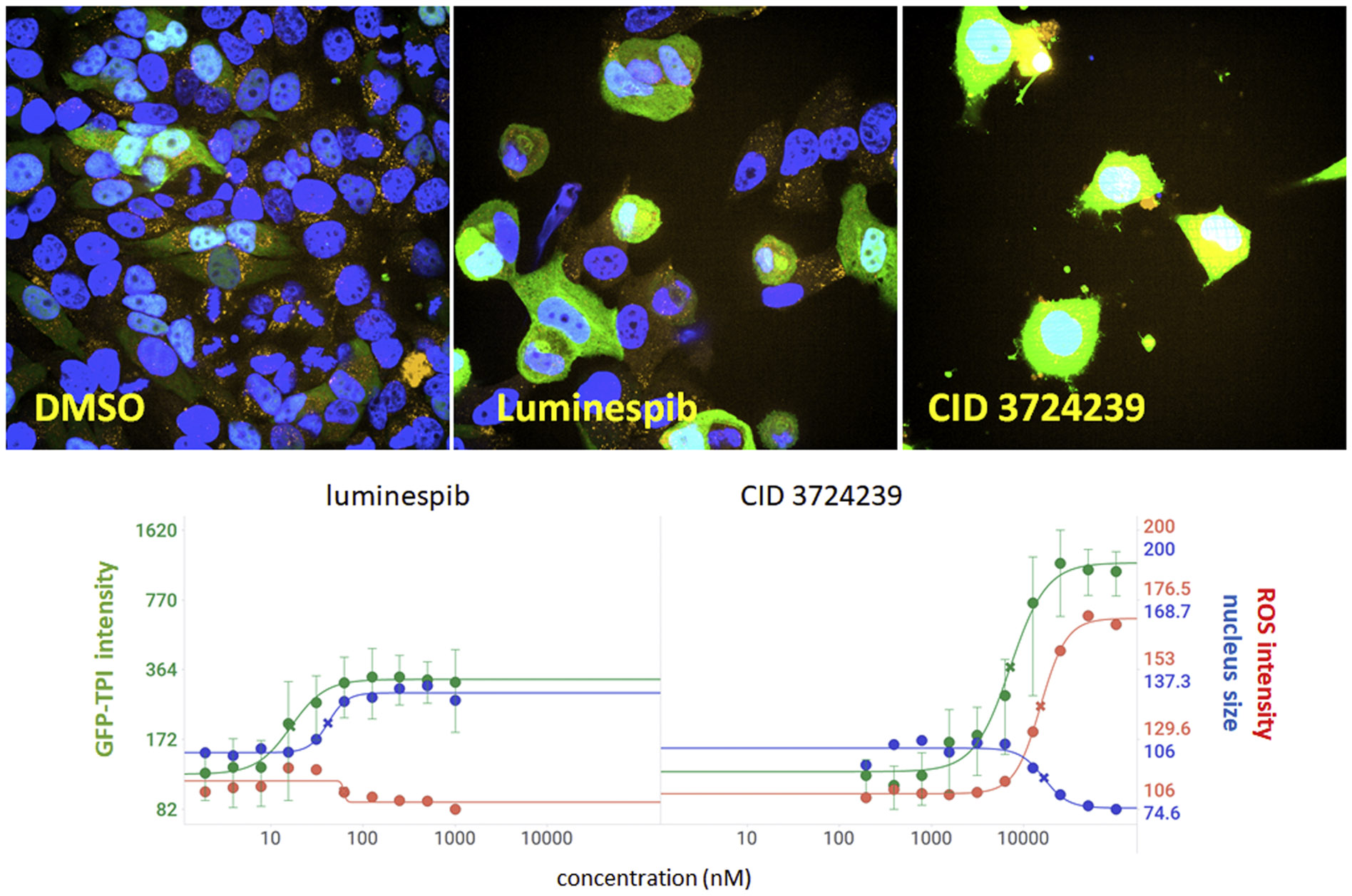
Appearance and behavior of the HeLa TPI^E105D-GF-HR^ cell line. Upper panel. Representative images of cells expressing mutant TPI-GFP (green) and a HyPerRed ROS biosensor (orange). Nuclei were counterstained with Hoechst 33342 (blue). Images were acquired on an OPERA Phenix Plus using a 63x water immersion objective in the DAPI, GFP, and Cy3 channel. Lower panel. Quantification of cellular response to test agents reveals desirable (elevated GFP in the absence of nuclear shrinkage and elevated ROS; luminespib) and undesirable activity profiles (elevated GFP correlating with nuclear shrinkage and elevated ROS; CID 3724239). Y-axes show percent increases compared with vehicle-treated cells. Data points are the average of at least three independent biological repeats ± SD. Curves were fitted to a four parameter logistic regression. Green, GFP-TPI levels, red, ROS, blue, nucleus size. Images were subjected to identical linear bitmap stretch for visibility.

We had previously reported the generation of HEK293 cells expressing a GFP-tagged mutant TPI protein [9]. Whereas this cell line permitted small scale high-content screening, it was found that loss of cell attachment during treatment and processing impeded its utility for large-scale HTS. We therefore generated a *HeLa TPI^E105D-GFP^* cell line, which was more resistant to cell loss during processing. Additionally, we incorporated a HyperRed-based biosensor for reactive oxygen species (ROS) [10]. Because ROS levels are the most reliable indicator for clinical toxicity [21], inclusion of the HyPerRed biosensor enables identification of compounds with potential clinical liabilities early in the screening process. Fluorescence micrographs in Fig. 1 illustrate the appearance of the *HeLa TPI^E105D-GFP-HR^* cells and their response to positive control compounds. Mutant TPI-GFP levels in un-treated cells are low and highly variable, with little to no detectable ROS (Fig. 1, DMSO). Luminespib, an inhibitor of heat shock protein 90 that was previously shown to increase mutant TPI levels [9,22], caused a three-fold increase of TPI-GFP that is most pronounced in the cytoplasm; its EC_50_ was 28 nM. The compound also slightly enlarged nuclei (by ~30 %), but did not show signs of overt toxicity (nuclear shrinkage, elevated ROS, or visually apparent cellular morphology changes). In contrast, CID 3724239, an arbitrarily chosen promiscuous, cytotoxic, alkaloid-like compound (https://pubchem.ncbi.nlm.nih.gov/compound/3724239) contained in the Cumbre pilot library caused pronounced cell loss, cellular morphology changes, and elevated ROS that correlated with GFP intensity. TPI-GFP, nuclear morphology, and ROS levels were quantified by high-content image analysis, and revealed activity/toxicity profiles for desirable and undesirable compounds (Fig. 1, graphs).

### Assay optimization.

We next optimized the *HeLa TPI^E105D-GFP-HR^* assay to accepted HTS performance [23]. To reduce heterogeneity in TPI-GFP expression, cells were first sorted by flow cytometry to select a population with intermediate GFP levels. We then examined cell density and passage dependence, length of compound treatment, signal stability during nuclear staining, and automation compatibility in live vs. fixed cells (Figure S1). Surprisingly, cell fixation dramatically increased assay variability, necessitating a live cell screen. Final assay conditions were 4,000 cells/well, two-day incubation with test agents, up to seven hours incubation in Hoechst 33342, imaging live on the OPERA Phenix Plus followed by automated image analysis in Harmony. The most robust readout was the total GFP intensity in the cytoplasm, which was set as the primary hit selection criterion and was evaluated for passage dependence in a multi-day variability assessment using full 384-well microplates each of positive and negative controls at different passages in culture on four different days (Table 1). The assay did not perform well immediately after thawing cells, but met accepted HTS criteria and performed with the same level of statistical significance in subsequent independent trials for at least 11 passages in culture.

### Assay validation.

**Fig. 2.**
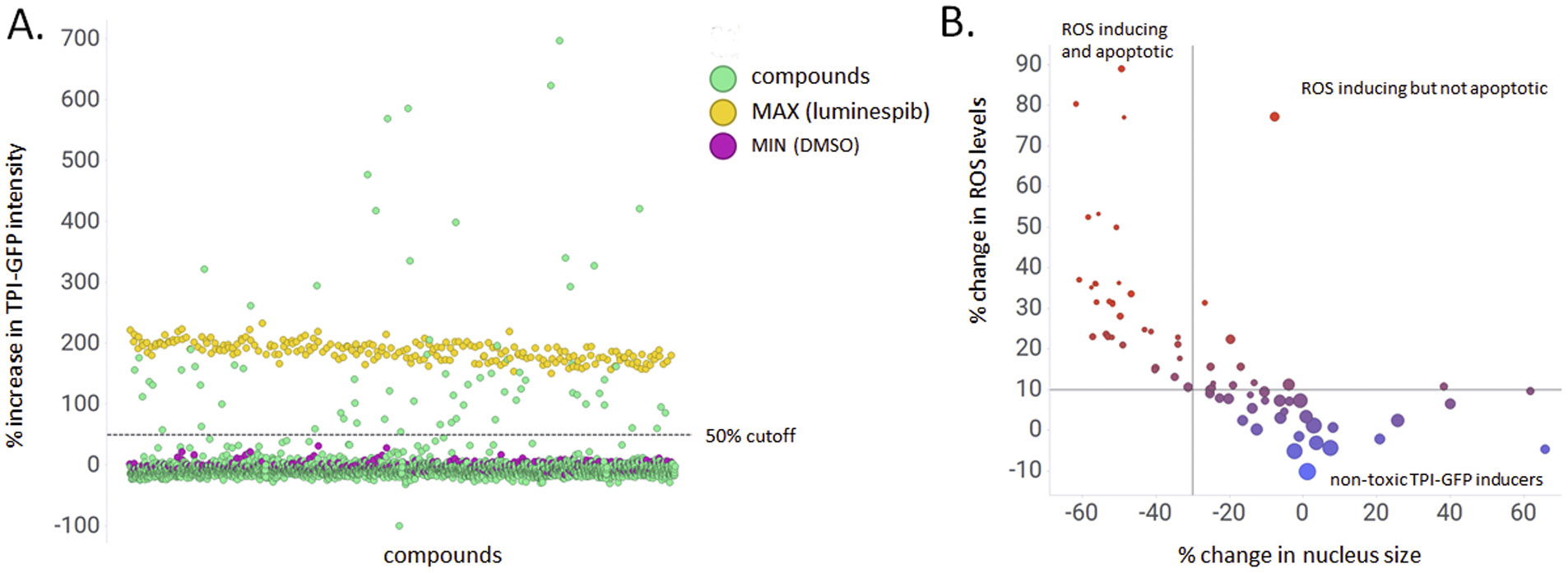
Hit selection paradigm developed from the pilot library screen. HeLa TPI^E105D-GFP-HR^ cells were treated with library compounds for 48 h, stained with Hoechst 33342, and imaged live on the OPERA Phenix Plus. TPI-GFP, nucleus size, and ROS expression were quantified using Harmony 5.1. A. Primary screen performance. Compounds that increased TPI-GFP by at least 50 % were scored as hits. Red, DMSO (MIN) controls, yellow, luminespib (MAX) controls, green, compounds. B. Seondary gates for cellular toxicity of primary screen hits. Evaluation of nucleus size as a proxy of apoptosis and ROS levels reveals different classes of hits. Size of data points additionally reflects number of cells/well (larger symbols representing more cells). ROS and apoptosis, as expected, correlate with loss of cells. Compounds that increased TPI-GFP without cellular toxicity were selected for confirmation in dose-response.

Using the optimized *HeLa TPI^E105D-GFP-HR^* assay, we then screened the Cumbre library, a collection of 2,560 structurally diverse small molecules for their ability to increase levels of mutant TPI-GFP. The primary screen was in live cells and performed to accepted HTS criteria (average Z’ 0.70 ± 0.02). Compounds that increased GFP by >50 % were scored as primary hits (Fig. 2A). This yielded 70 compounds (2.74 % hit rate). To reduce the number of hits, additional gates were set for nuclear morphology and ROS. Visual inspection of archived images revealed that a reduction of nucleus size by >30 % correlated with apoptotic cells, and an ~10 % increase in ROS was clearly discernible as elevated ROS. This final filtering strategy gave 28 hits (a 1.1 % hit rate; Fig 2B).

### Dose-response confirmation in HeLa *TPI^E105D-GFP-HR^* cells.

**Fig. 3.**
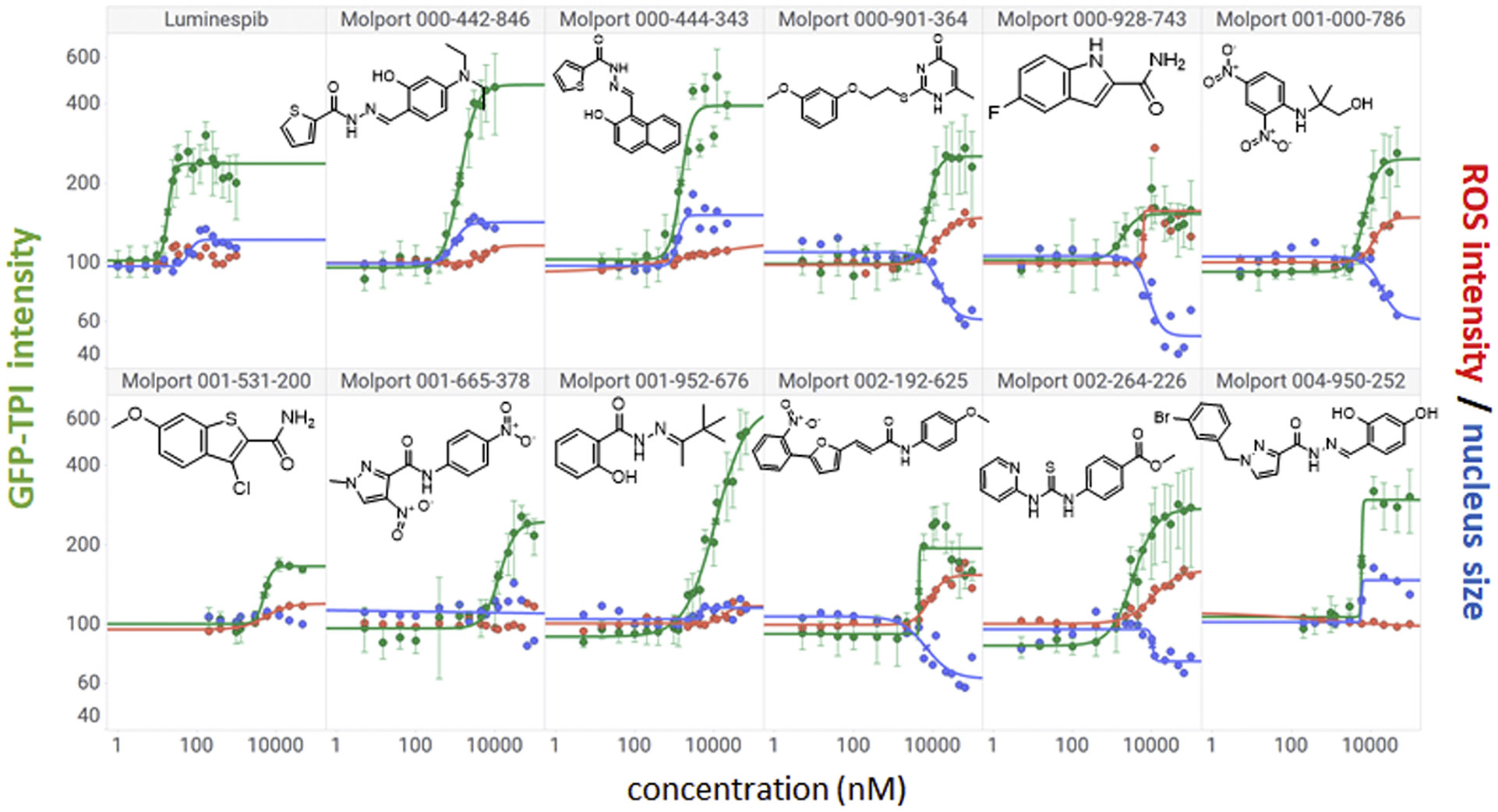
Dose-response confirmation of pilot library hits in HeLa TPI^E105D-GFP-HR^ cells. Hits from the pilot screen were tested in ten-point, two-fold concentration gradients and analyzed by high-content analysis for dose-dependent increases in mutant TPI-GFP (green) and markers of cellular toxicity (nucleus size (blue) and ROS (red)). Eleven compounds showed full sigmoidal TPI-GFP curves. Four agents had favorable activity/toxicity profiles, shared a common chemical structure element, and were selected for further confirmation in TPI-Df patient fibroblasts. Data are the averages of up to 12 independent biological repeats ± SD. Y-axes show percent increases compared with vehicle-treated cells.

From the 28 primary hits, 25 were commercially available and ordered from Molport (https://www.molport.com). The list was supplemented with five additional, structurally similar compounds that did not register as primary hits, for a total of 30 compounds that were subjected to ten-point, two-fold concentration gradients in HeLa *TPI^E105D-GFP-HR^* cells (Table S1). Fourteen compounds (37 %) showed at least some activity (Table S1), and 11 had full sigmoidal dose-response curves (Fig. 3). Five of those (MolPort-000–901–364, 000–928–743, 002–192–625, and 002–264–226) showed undesirable toxicity profiles, namely condensed nuclei and elevated ROS that mirrored TPI expression (cf. Fig. 1). Visual confirmation was used to remove compounds that showed ambiguously elevated TPI-GFP levels, significant changes in cellular morphology, or contained imaging artifacts. Most of these also did not pass cheminformatic filters using the FAFDrugs4 server at https://fafdrugs4.rpbs.univ-paris-diderot.fr/ [16]. The compound class that consistently presented with full dose-response curves and desirable activity/toxicity profiles were carbohydrazides (MolPort-000–442–846, 000–444–343, 001–952–676, and 004–950–252), which were overrepresented in the hit list and showed differences in potency, suggesting possible structure-activity relationships (SAR). Evidence for SAR was further supported by four carbohydrazides that lacked TPI-inducing activity (Molport-001–897–405, 004–950–612, 004–951–042, and 004–951–015; Table S1).

### Activity in TPI-Df patient cells.

**Fig. 4.**
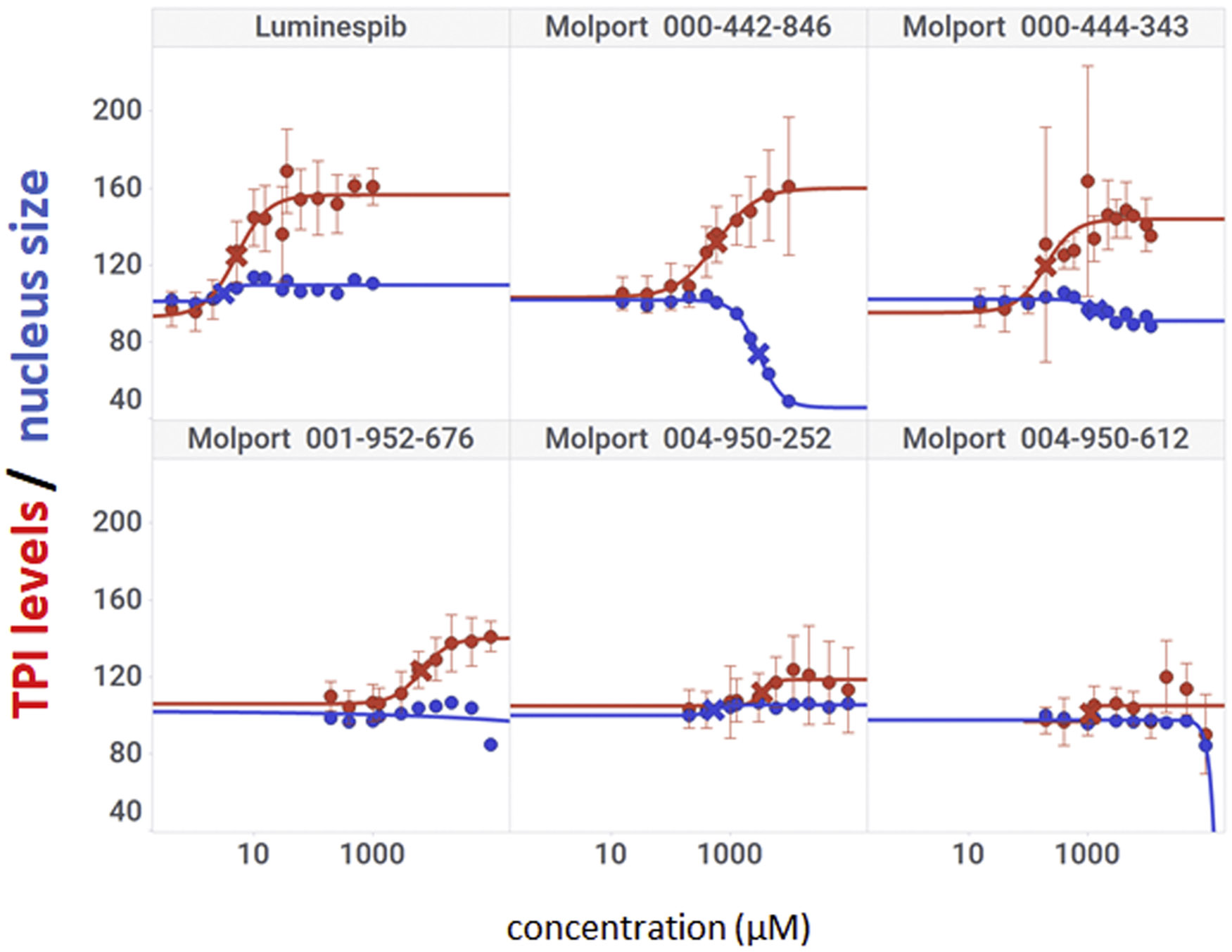
Activity and toxicity of carbohydrazides in TPI-Df patient fibroblasts. FB303 (TPI^E105D/E105D^) mutant TPI fibroblasts were treated with concentration gradients of selected carbohydrazides from the Cumbre pilot screen. TPI was visualized and quantified by indirect immunofluorescence; nuclear morphology by Hoechst 33342 counterstaining. Red, TPI levels; blue, nucleus size. Y-axis shows percent increases compared with vehicle-treated cells. Data points are mean ± SD from at least three independent biological repeats.

We then tested five carbohydrazides in TPI-Df patient fibroblasts (Fig. 4). Whereas compounds that showed full dose-curves in HeLa cells also increased endogenous mutant TPI in patient cells, we found some liabilities. At higher concentrations, Mol-Port-000–442–846 and 000–444–343 formed insoluble fluorescent precipitates (not shown); MolPort-000–442–846 also caused changes in nuclear morphology. Molport-004–950–252 and 001–952–676 were non-toxic but not particularly potent. Additionally, carbohydrazides can hydrolyze to hydrazides and aldehydes, which could be potentially harmful [24]. Therefore, the carbohydrazides were not prioritized for further study in animal models but set aside as potential tool compounds to investigate mechanisms of TPI stabilization. The pilot screen provided, however, important lessons with regard to scaling up to HTS format. First, it provided guidance for hit selection and follow-up. Second, hits that confirm visually and show a relevant dose-response curve in HeLa cells would likely confirm in TPI-Df patient cells. Third, visual inspection of archived images followed by selection of hits that clearly elevated GFP without any changes in cellular, nuclear morphology or ROS would dramatically reduce the number of hits that fail in dose-response. Lastly, examination of chemical structures suggested that in HTS a cheminformatics filter would be necessary to reduce the number of compounds with unfavorable physicochemical properties.

### Primary HTS.

**Fig. 5.**
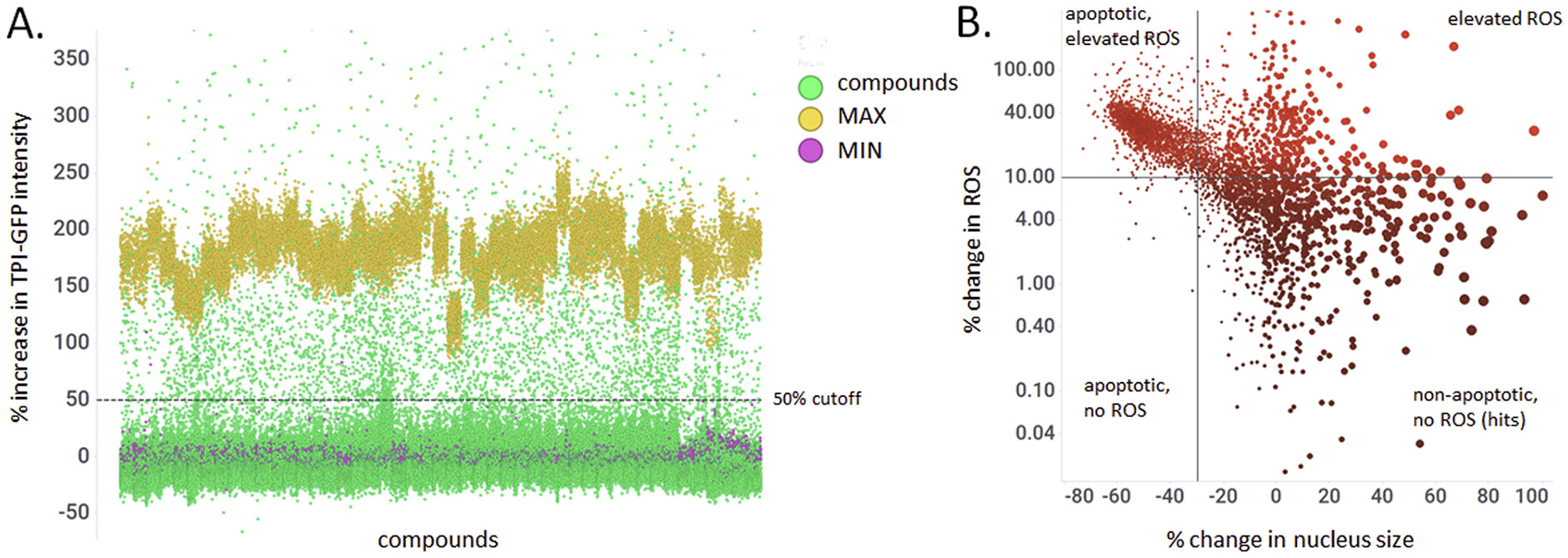
High-throughput screening of the MLSMR collection. HeLa TPI^E105D-GFP-HR^ cells were treated with library compounds for 48 h, stained with Hoechst 33,342, and imaged live on the OPERA Phenix Plus. TPI-GFP, nucleus size, and ROS expression were quantified using Harmony 5.1. Hit selection followed that described previously (Fig. 2).

Based on the results from the pilot screen, we proceeded to perform a full-scale HTS using the NIH MLSMR compound collection of 220,700 agents, applying the numerical hit selection criteria established in the pilot screen (Fig. 5). The screen consisted of 696 plates, which were processed in 48 runs of ~5,000 compounds each. The average Z’ across all plates was 0.72 ± 0.06, with no plate failures. This yielded 1,863 primary hits (0.25 % hit rate), which were visually examined in archived scan images for fluorescent artifacts, clearly and unambiguously elevated GFP levels, changes in cellular or nuclear morphology, and elevated ROS. This yielded 562 compounds that convincingly presented with elevated TPI-GFP without changes in nuclear and cellular morphology, which were subjected to cheminformatics analyses [16] to eliminate agents with PAINS flags, potential liabilities for future development, and unwanted structural elements such as multiple chiral centers. The final hit list comprised 83 agents (0.04 % hit rate) of which 60 were available and repurchased from commercial vendors (Table S2).

### HTS dose-response.

**Fig. 6.**
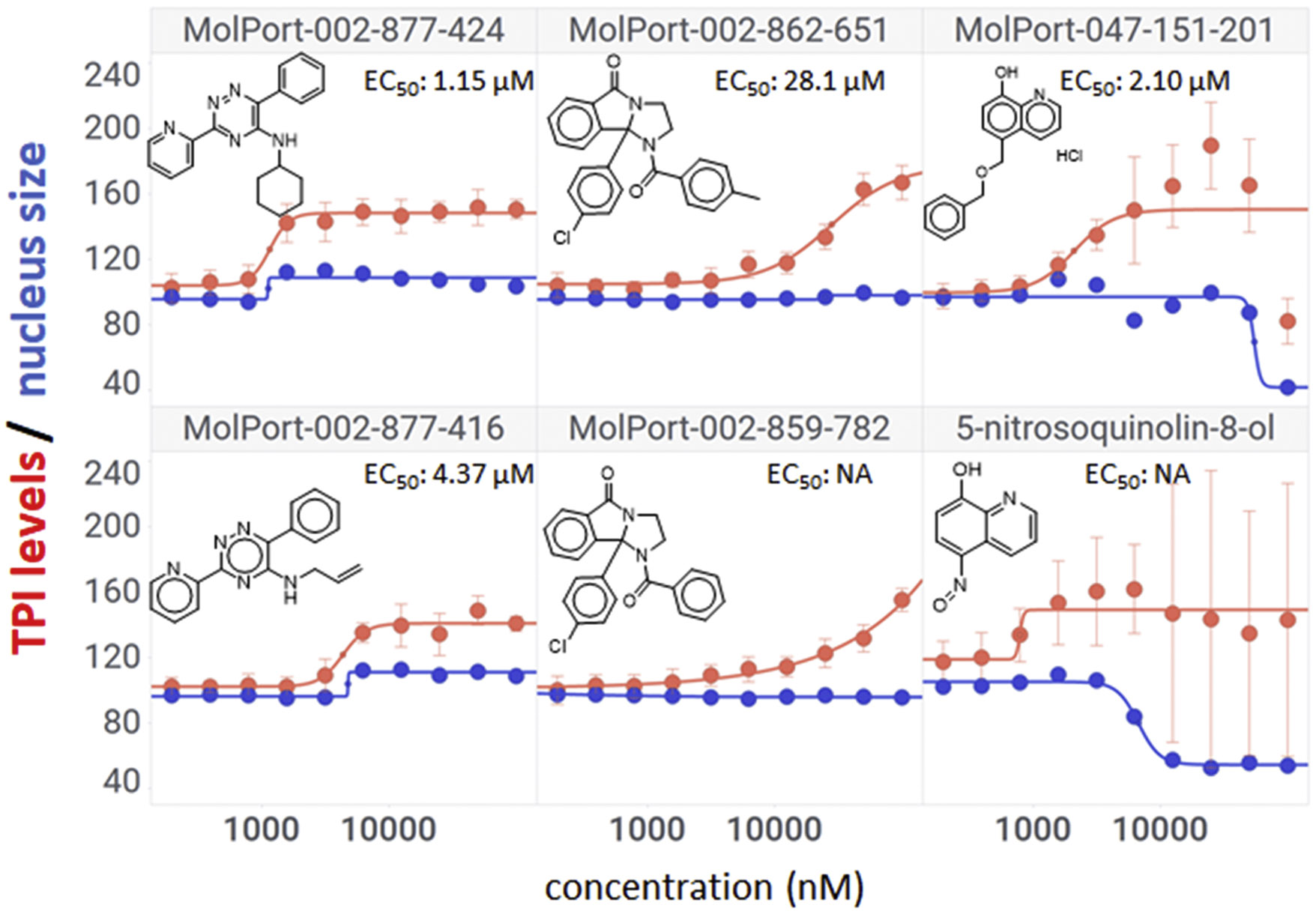
Selected hits from HTS in FB303 patient cells. Compounds were chosen based on repeat activity in patient cells, potency, lack of toxicity, and presence of analogs. Data points represent the mean ± SD from at least three independent biological repeats.

**Fig. 7.**
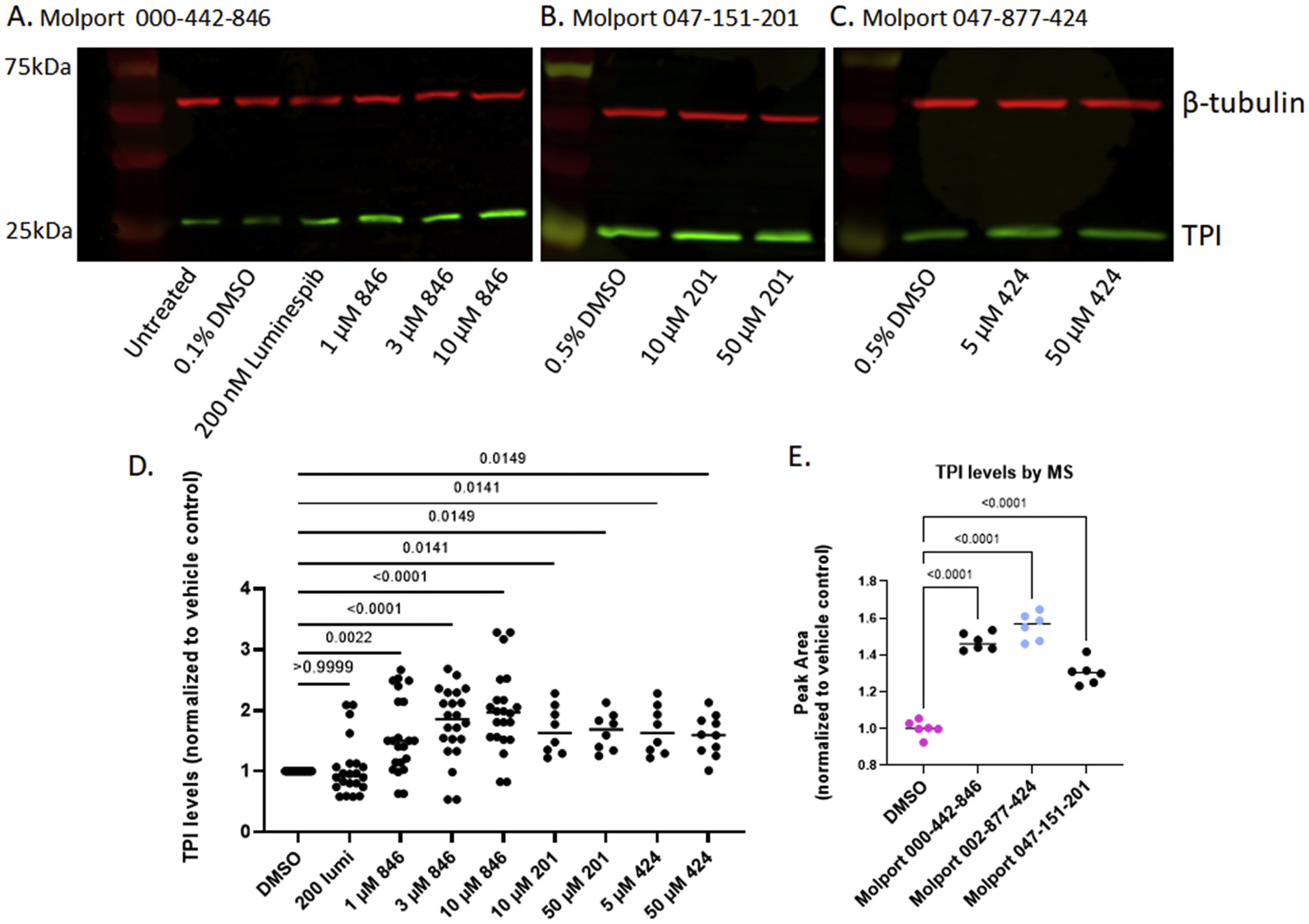
Confirmation of elevated TPI levels in FB303 patient fibroblasts by Western blot and MS. Blots are representative examples of multiple independent experiments. Graphs in D. are the aggregate of the indicated number of biological repeats and show the TPI/tubulin ratios normalized to DMSO. p-values were obtained by nonparametric analysis with Kruskal Wallis correction. Graph in E. is from a single experiment with 6 technical replicates. p-values were by nonparametric analysis with Kruskal Wallis correction.

The 60 commercially available compounds were subjected to 10-point, two-fold dose-responses in the stable HeLa *TPI^E105D-GFP-HR^* cell line, and evaluated for well-behaved dose-response curves, potency, toxicity, and visual appearance, as described for the pilot library. The compounds that consistently met those criteria fell into three different structural classes: 1,2,4-triazines, imidazo-[2,1*a*] isoindol-5-ones, and 8-hydroxyquinolines. All these compounds repeated in TPI-Df fibroblasts, with significant loss of activity for the imidazo-[2,1a] isoindol-5-ones and toxicity for 5-nitroso quinoline-8-ol (Fig. 6). The most potent 1,2,4 triazine and 8-hydroxyquinoline were then further confirmed in Western blots, together with the most promising compound from the Cumbre library (Fig. 7A-D). The immunofluorescence assay was additionally validated by a second orthogonal assay (mass spectrometry) (Fig. 7E).

### Structure activity relationship studies.

Limited SAR studies on the carbohydrazides (Fig. 3, Fig. 4 and Table S1), 1,2,4 triazines, and 8-hydroxyquinolines (Table 2) confirmed the activity of the respective scaffolds and showed differences in potency, toxicity, and solubility. Notably, Molport 047–151–201 had widely varying responses between batches and between experiments.

### Possible mechanism(s) for TPI induction.

**Fig. 8.**
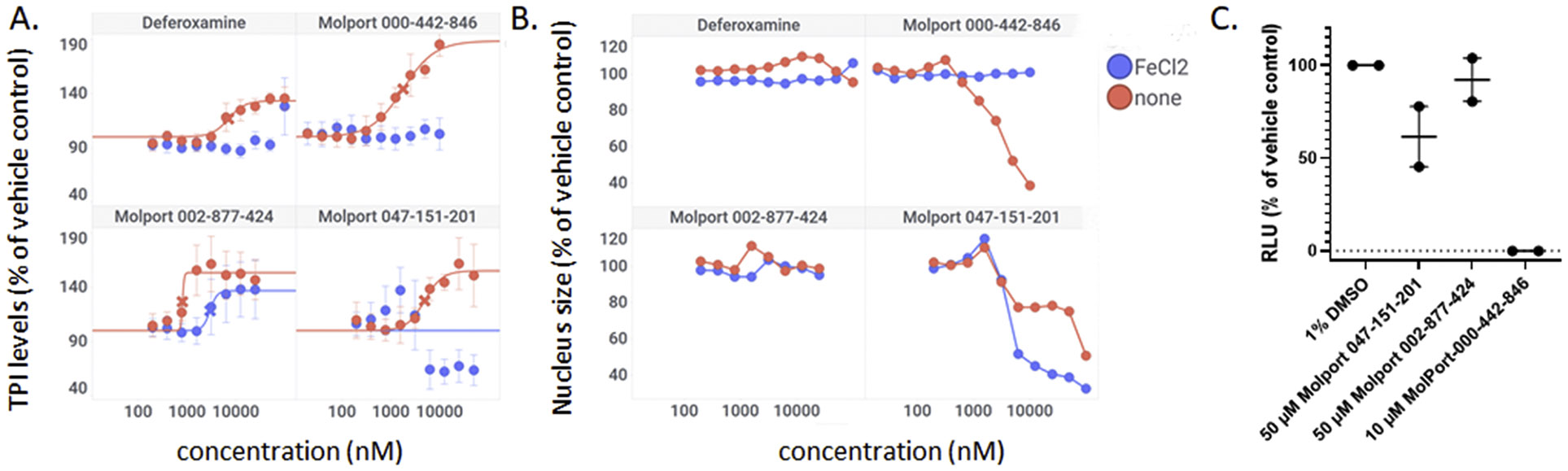
Metal dependence and proteasome inhibition in FB303 TPI-Df patient cells. A, B. Cells were treated with the indicated concentrations of compounds for two days and immunostained with an anti-TPI/Cy3 secondary antibody pair. A. Activity of hits in the absence (red) and presence (blue) of 100 μM ferrous chloride. B. Corresponding cytotoxicity assessments. Y-axes show percent increases compared with vehicletreated cells. C. Proteasome inhibition in the presence of vehicle (DMSO), Molport 000–442–846 (10 μM), 047–151–201 (50 μM), and 002–877–424 (50 μM). RLU, relative luminescence intensity, corrected for background and normalized to vehicle control. Data points represent median ± range from two independent repeats with 6 and 16 technical replicates, respectively.

SAR studies showed that only carbohydrazides with 2-hydroxy-benzylidene moieties were active, as were 8-hydroxyquinolines with a free hydroxyl group. This indicated that metal chelation could be a mechanism for TPI induction. To test this, we titrated selected hits and a known iron-chelating agent (deferoxamine) in the presence of ferrous chloride (FeCl_2_), which is expected to abolish activity of chelators. Deferoxamine was able to induce TPI in a dose-dependent manner and excess ferric chloride completely inhibited the activity of deferoxamine (Fig. 8A, B). Also, the carbohydrazide 000–442–846 and the 8-hydroxyquinoline 047–151–201 behaved similarly (Fig. 8A, B). Molport 002–877–424, however, was only partially affected, suggesting it might have activites not related to metal chelation (Fig. 8A, B). We therefore tested all agents in a proteasome assay and found that 000–442–846 strongly inhibited the proteasome but 002–877–424 and 047–151–201 did not (Fig. 8C). Interestingly, the effect of ferrous chloride on cellular compound toxicity was varied. Whereas toxicity of deferoxamine and 002–877–424 was unaffected, 047–151–201 toxicity was accentuated, and that of 000–442–846 was abolished (Fig. 8B).

### Preliminary in vivo studies.

Because of the above-mentioned short-comings of Molport 000–442–846 and 047–151–201, we selected Molport 002–877–424 as our lead compound. To prepare for future testing of in vivo activity in our mouse model of TPI-Df [25], we performed preliminary PK and tissue distribution studies of 002–877–424 in mice. The compound was resynthesized by Enamine on a gram scale and identity and purity verified by LC/MS. Following intraperitoneal administration at 10 mg/kg, the compound rapidly reached its peak plasma concentration (Cmax) of 9.91 μg/mL (~ 30 μM) within 30 min (Tmax) post dose. After that, its plasma concentration declined with a last measurable concentration of 240 ng/mL at 24 h and a moderate terminal half-life (t1/2) of 4.64 h (Table S3). The total systemic exposure (AUCinf) was 66.0 h*μg/mL (Table S3). In the tissue distribution study, the compound accumulated preferentially in liver and kidney, reached moderate levels (~1 – 1.6 μM) in muscle, and was moderately brain-penetrant, with mean tissue to plasma ratios of up to 0.134 (Table S4). A molecule is commonly deemed CNS penetrant if its brain-to-plasma concentration ratio (Cb:Cp) is >0.04 using nonperfused brain tissue as cerebral blood volume approximates 4 % of total brain volume [26], although this is only an approximation, and free drug levels need to be determined experimentally (for example by micro-dialysis). Nonetheless, a brain-to-plasma ratio of 0.1 is deemed moderately brain penetrant [27]. The ability to enter relevant target tissues is important for future PK/PD studies and, ultimately, potential application to TPI-Df patients.

## 4. Discussion

TPI deficiency is a devastating, incurable childhood disease for which no treatments exist. The underlying cause of the disease are loss-of-function missense mutations of the *TPI1* gene that cause instability of the TPI protein, reducing its levels and impairing glycolysis. Mutant proteins retain significant function; therefore, we hypothesized that agents that increase mutant TPI levels could ameliorate disease symptoms. To that end, we had previously described the development and validation of an image-based, high-content screen for GFP-tagged mutant TPI and shown that it could discover small molecules that increase levels of mutant TPI in cell culture models of TPI-Df. Here, we expanded these efforts to a ~225,000 compound lbrary, using a HeLa cell line that expresses GPP-tagged mutant TPI and a red ROS biosensor. The screen, together with cheminformatics analysis and a series of functional and mechanistic secondary assays, identified three promising structural scaffolds that increased mutant TPI in TPI-Df patient fibroblasts without overt cytotoxicity. Mechanistically, all compouds had metal-chelating activities, but only one of them inhibited the protea-some. A lead from the most promising series, Molport 002–877–424, a 1,2,4-triazine, was chosen for animal studies.

### Potential mechanisms for TPI induction.

All prioritized agents had iron-chelating activities. Excess ferrous chloride reduced the activity of most agents, but their extent of iron-induced toxicity varied. Whereas 8-hydroxyquinoline toxicity was accentuated, that of the carbohydrazide 000–442–846 was abolished, and the 1,2,4-triazine 002–877–424 was unaffected. This indicates that TPI levels are modulated by iron but that iron chelation is not necessarily detrimental to cell survival. The reasons for the differential reponse to excess iron are unclear but suggest the compounds may be targeting different iron-dependent cellular functions. Further studies are needed to investigate the details of iron chelation-mediated stabilization of TPI, including limitations arising from variability in iron dependence.

### Proteasome inhibition as a mechanism for TPI induction.

Prior work from our laboratories has shown that proteasome inhibitors increase TPI levels in an invertebrate model of TPI-Df [22,28]. That class of agents is being developed as anticancer agents, and therefore, is unlikely to be beneficial for a chronic disease where patients are already health-compromized. However, since iron chelation is not known to inhibit the proteasome, 000–442–846 could affect both synthesis and degradation of mutant TPI through iron chelation and through (iron--independent) proteasome inhibition. Additional mechanism of action (MOA) and target identification studies are needed to answer this question and are ongoing. Nonetheless, the agents identified may prove beneficial as tool compounds due to the magnitude of their response, despite an incomplete understanding of their MOA.

### Magnitude of response needed to anticipate clinical response.

Disease severity in patients correlates with the extent of the reduction in TPI protein [2]. Therefore, any significant increase in TPI protein could provide an amelioration in symptoms. Moreover, the disease is fully recessive and heterozygote patients with ~50–60 percent reduction in TPI protein levels and activity are completly asymptomatic ([1,2]; and unpublished data). Therefore, there is significant reserve TPI capacity creating a disease threshold that is incompletely understood. This is also complicated by the fact that TPI has additional functions outside of glycolysis, some of which reportedly do not require isomerase activity [29]. Nonetheless, based largely upon asymptomatic heterozygous patient data, it is a reasonable estimate that an ~20–30 % increase would be needed to completely ameliorate symptoms in most TPI-Df patients. The lead compounds described here are producing an ~ 50 % increase in patient cells, suggesting they could significantly ameliorate disease severity. Of course, how effective a small molecule therapy is for TPI-Df will depend on more than just the magnitude of the effect but also on total exposure to and tissue distribution of the drug, as well as when the therapy is initiated in the disease course. It is possible that some of the symptoms observed could become irreversible if treatment is not initated early in the disease course. Data from a mouse model of TPI-Df suggest locomotor symptoms likely develop due to severe muscle wasting, neurologic dysfuction, and neuropathology that may become irreversible if treatments are delayed [8,25].

### New directions emanating from the high throughput screen.

The triazine hits had previously been identified during a screen for hypoxia-inducible factor 1 (HIF1) alpha modulators [30]. We hypothesized that those could be beneficial in TPI-Df for several reasons. TPI-Df is a defect in glycolysis and one of the earliest symptoms of TPI-Df is hemolytic anemia. HIF1 alpha induces essentially all glycolytic enzymes, shifting cellular metabolism from oxidative phosphorylation to glycolysis [31], and TPI is a known target gene for HIF1 alpha [32]. Furthermore, HIF1 alpha activation increases erythropoietin (EPO), and thus, production of red blood cells. Several activators of HIF1 alpha, which are unrelated to the triazines, are in clinical use. These compounds stabilize HIF1 alpha by inhibiting prolyl hydroxylases (PHD). HIF1 alpha is degraded by the ubiquitin system after proline hydroxylation [33-35]. The reaction is catalyzed by a family of dioxygenases termed prolyl hydroxylases (PHD) [36,37], and is iron-dependent [38]. Early PHD inhibitors were metal chelators and some have been tested in the clinic (e.g., deferoxamine). An analog from the triazine series (ML-228, Table 2) was tested by another group and shown to be iron-dependent as well [30]. Whereas the activity of ML-228 was completely abolished by ferrous chloride [30], that of analog 002–877–424 was only partially affected, suggesting that there could be subtle differences in the mechanism of action within the 1,2,4-triazine series. Going forward, it will be important to investigate the extent to which HIF1-alpha affects TPI levels, for example by using HIF1 alpha null cells or genetic knockouts.

### Outlook and perspective.

PHD inhibitors are in clinical use for treatment of hemolytic anemia in patients with chronic kidney disease [39,40]. Because anemia is one of the earliest symptoms of TPI-Df, this opens the exciting possibility of repurposing PHD inhibitors for TPI-Df. We are currently testing this hypothesis by evaluating several clinically used PHD inhibitors in our in vitro and in vivo TPI-Df models, with the ultimate aim to advance a treatment for patients with this devastating, incurable childhood disease.

## Supplementary Material

1

2

3

4

5

Supplementary material associated with this article can be found, in the online version, at doi:10.1016/j.slasd.2025.100278.

## Figures and Tables

**Table 1:** Multi-day assay variability and passage dependence of the HeLa *TPI^E105D-GFP-HR^* assay. MIN and MAX data are total GFP intensities in the cytoplasm.

passage after thaw	1	3	9	11
MIN (DMSO)	1,256,111	1,705,103	1,795,742	1,671,060
MAX (luminespib)	2,193,591	5,306,517	5,406,459	5,340,642
S/B	1.75	3.11	3.01	3.20
% CV	14 %	6 %	4 %	4 %
Z-prime	−0.42	0.59	0.65	0.50
N	384	384	768	768

**Table 2:** SAR in FB303 patient fibroblasts for selected hit series from the MLSMR high-throughput screen.

Compounds	Pubchem CID	Structure	EC_50_ (μM) Mean ± SD (n)	Notes
1,2,4 triazines				
MolPort–002–877–424	6404647	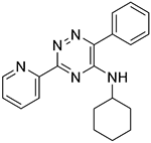	0.96 ± 0.31 (14)	
MolPort–002–877–416	6413024	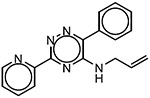	4.00 ± 0.77 (4)	
	6404645	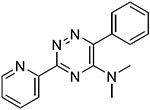	6.95 ± 4.74 (2)	
ML228 [30]	46742353	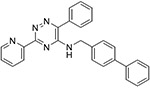	0.34 ± 0.05 (4)	Solubility problems above 3 μM
8-hydroxyquinolines				
Molport 047–151–201	6603466	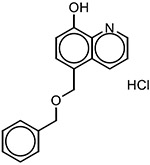	4.36 ± 4.55 (15)	High variability between experiments; notable toxicity above 50 μM
5-nitroso quinoline-8-ol	19103	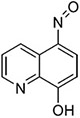	No plateau; some activity above 5 μM	notable toxicity above 5 μM
8-hydroxyquinoline	1923	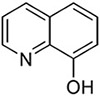	3.37 ± 2.46 (3)	
8-methoxyquinoline	70310	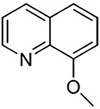	>> 100 (3)	

## Abbreviations:

Cy3: indocarbocyanine with three-carbon linker
DHAP: dihydroxyacetone phosphate
GFP: green fluorescent protein
HCS: high-content screening
HTS: high-throughput screening
HIF1: hypoxia-inducible factor 1
IM/MS: ion mobility/mass spectroscopy
LC/MS: liquid chromatography/mass spectrometry
MeCN: acetonitrile
MGO: methylglyoxal
MLSMR: molecular libraries small molecule repository
PK: pharmacokinetics
ROS: reactive oxygen species
TPI: triose phosphate isomerase
TPI-Df: TPI deficiency
