# Supplementary material for: Discovery and validation of small molecule stabilizers of mutant triose phosphate isomerase (TPI) as potential lead candidates for TPI deficiency
Source: SLAS Discov. Author manuscript; Available in PMC 2025 Dec 16. (PMC12707346; doi:10.1016/j.slasd.2025.100278)
Supplement: 3 [file NIHMS2122050-supplement-3.docx]

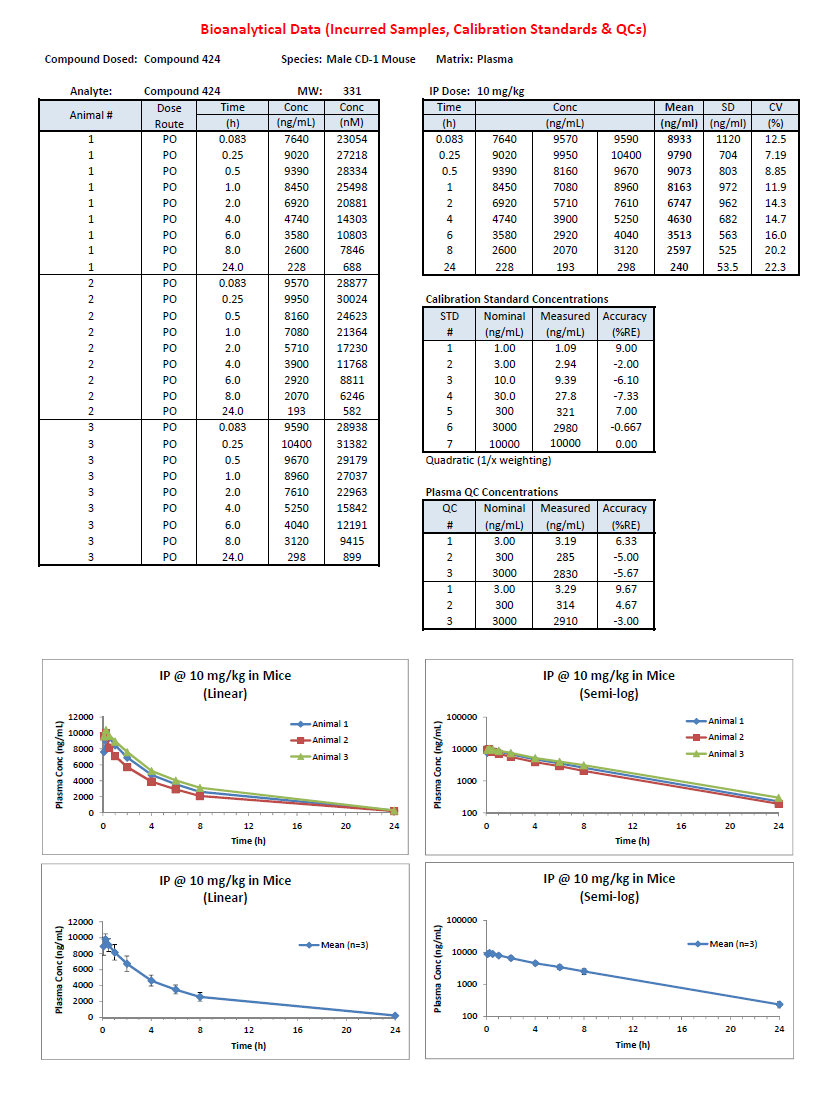

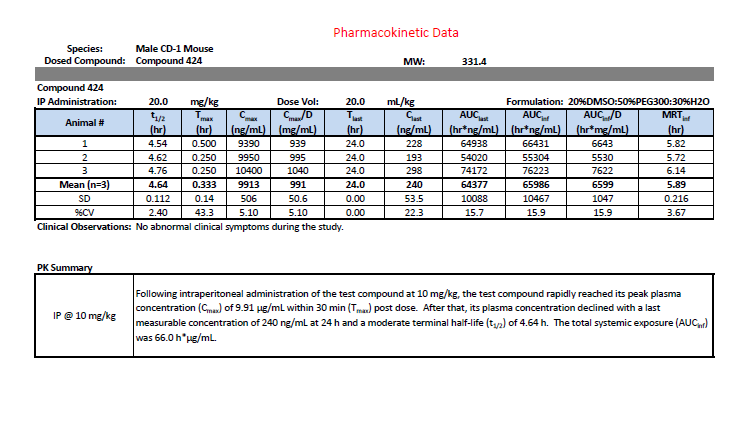
**Table S3. Pharmacokinetic data for Molport 002-877-424 in CD-1 mice.**

Pharmacokinetic data were generated by Touchstone biosciences; detailed methodology can be found in the Materials and Methods section and in (Vollmer, Liu et al. 2025).

Vollmer, L. L., F. Liu, B. Nmezi, G. R. Bey, N. Herdman, T. Y. Shun, A. Gough, R. Liu, P. Wipf, T. R. Lezon, Q. S. Padiath and A. Vogt (2025). "A high throughput, high content screen for non-toxic small molecules that reduce levels of the nuclear lamina protein, Lamin B1." Sci Rep **15**(1): 7314.
