# Supplementary material for: Discovery and validation of small molecule stabilizers of mutant triose phosphate isomerase (TPI) as potential lead candidates for TPI deficiency
Source: SLAS Discov. Author manuscript; Available in PMC 2025 Dec 16. (PMC12707346; doi:10.1016/j.slasd.2025.100278)
Supplement: 5 [file NIHMS2122050-supplement-5.docx]

**Figure S1. Assay optimization.**
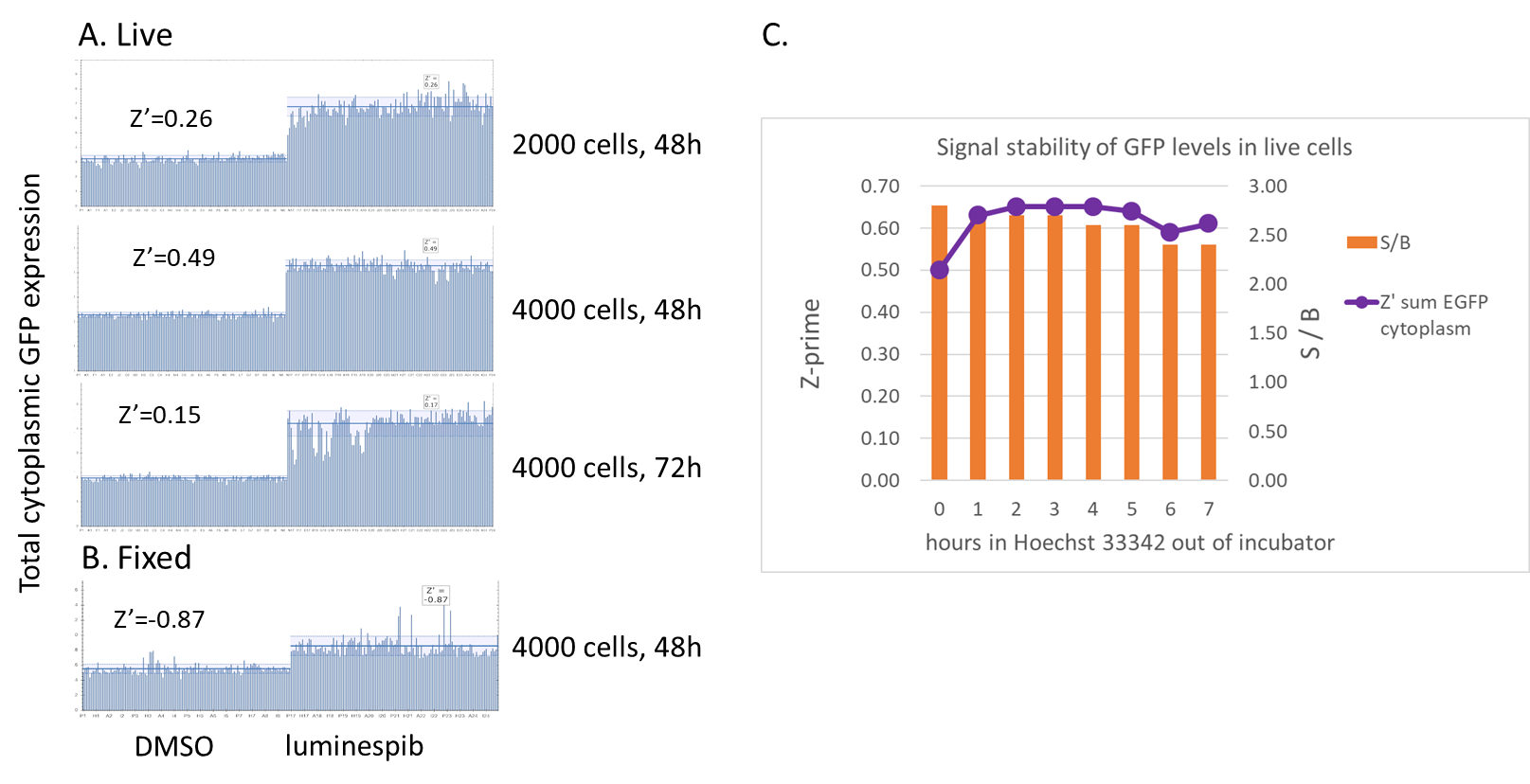


**Figure S1. Assay optimization.** Cells were treated with DMSO or luminespib (200 nM) for the indicated times and imaged on the OPERA Phenix plus using a 20x air objective. **A.** Cell density and length of incubation time in live cells show best performance at the 48h time point with 4000 cells/well. **B.** Formaldehyde fixation at the optimal time point dramatically reduced assay performance, necessitating a live cell screen. **C.** Stability of GFP signal in Hoechst 33342 nuclear stain at RT in air. To mimic conditions of live cell screening, cells were treated for 48 h with vehicle or luminespib and pre-stained with Hoechst 33342 nuclear stain for one hour. Plates were placed on a plate stacker and read on the OPERA Phenix plus every hour for 7 hours. The assay performed to HTS criteria for at least 7 h (Z’ values >0.5).
